# Central Sensitization of Mechanical Nociceptive Pathways Is Associated with a Long-Lasting Increase of Pinprick-Evoked Brain Potentials

**DOI:** 10.3389/fnhum.2016.00531

**Published:** 2016-10-20

**Authors:** Emanuel N. van den Broeke, Julien Lambert, Gan Huang, André Mouraux

**Affiliations:** Institute of Neuroscience, Université Catholique de LouvainBrussels, Belgium

**Keywords:** central sensitization, hyperalgesia, mechanical, pinprick, evoked potentials, brain

## Abstract

Intense or sustained nociceptor activation, occurring, for example, after skin injury, can induce “central sensitization,” i.e., an increased responsiveness of nociceptive neurons in the central nervous system. A hallmark of central sensitization is increased mechanical pinprick sensitivity in the area surrounding the injured skin. The aim of the present study was to identify changes in brain activity related to this increased pinprick sensitivity. In 20 healthy volunteers, increased pinprick sensitivity was induced using high frequency electrical stimulation of the forearm skin (HFS). Mechanical pinprick stimulation (64 and 90 mN) was used to elicit event-related brain potentials (ERPs). The recordings were performed before, 20 min after and 45 min after applying HFS. The contralateral non-sensitized arm served as control. Pinprick stimulation of 64 mN, but not 90 mN, applied in the area of increased pinprick sensitivity elicited a significant increase of a late-latency positive wave, between 300 and 1100 ms after stimulus onset and was maximal at midline posterior electrodes. Most importantly, this increase in EEG activity followed the time course of the increase in pinprick perception, both being present 20 and 45 min after applying HFS. Our results show that the central sensitization of mechanical nociceptive pathways, manifested behaviorally as increased pinprick sensitivity, is associated with a long-lasting increase in pinprick-evoked brain potentials provided that a 64 mN stimulation intensity is used.

## Introduction

Injury to the skin leads to increased pain sensitivity of the injured skin (“primary hyperalgesia”) but also of the surrounding *uninjured* skin (“secondary hyperalgesia”). A hallmark of secondary hyperalgesia is increased sensitivity to mechanical nociceptive stimuli such as pinprick stimuli (Raja et al., 1984; Ali et al., 1996; Magerl et al., 1998). The development of this increased pinprick sensitivity does not necessarily require tissue injury, as it can also be induced experimentally by activating nociceptors in a sustained and intense fashion, for example, using high frequency electrical stimulation of the skin (Henrich et al., 2015) or intradermal injection of capsaicin (LaMotte et al., 1991; Ziegler et al., 1999; Magerl et al., 2001). Importantly, the increase in pinprick sensitivity in the area of secondary hyperalgesia is thought to predominantly result from *central sensitization* (Baumann et al., 1991; LaMotte et al., 1991; Simone et al., 1991), defined by the International Association for the Study of Pain (IASP) as “*increased responsiveness of nociceptive neurons in the central nervous system to their normal or subthreshold afferent input.”*

In an attempt to explore the changes in brain activity related to this increased pinprick sensitivity, we recently recorded event-related brain potentials (ERPs) to mechanical pinprick stimulation of the skin before and after intradermal capsaicin injection (van den Broeke et al., 2015). We found that pinprick stimulation elicits both an early-latency negative-positive complex, maximal at the scalp vertex, and a later long-lasting positive wave, maximal at more posterior brain regions. When the pinprick stimuli were applied to the area of secondary hyperalgesia, *only* the magnitude of this late positivity was significantly increased.

However, we also showed recently that, in addition to inducing increased pinprick sensitivity, HFS also induces an enhancement of the brain responses to thermal and innocuous tactile stimuli (van den Broeke and Mouraux, 2014a,b). These after-effects were observed 20 min after applying HFS, but were no longer present 45 min after HFS. This was in striking contrast with the robust increase in pinprick sensitivity that was observed both 20 and 45 min after HFS, and is known to last up to several hours (Pfau et al., 2011). Taken together, this indicates that the intense and sustained activation of nociceptors triggers distinct mechanisms: nociceptive-specific mechanisms generating a long-lasting increase in pinprick sensitivity and unspecific mechanisms generating a short-lasting enhancement of the brain responses to a variety of sensory stimuli.

Therefore, in order to determine whether the increase in magnitude of the late positive wave of pinprick-evoked potentials (PEPs) is somehow related to the phenomenon of mechanical hyperalgesia, it is crucial to demonstrate that this increase in brain response follows the same time course as the increase in pinprick sensitivity, i.e., that it is also present 45 min after applying HFS. This constituted the aim of the present study.

## Materials and methods

### Participants

Twenty healthy volunteers took part in the experiment (8 men and 12 women; aged 20–27 years; 22.6 ± 2.0 years [mean ± SD]). The experiments were conducted according to the Declaration of Helsinki. Approval for the experiment was obtained from the local Ethical Committee (Hospital and Departmental Ethics Committee, Saint-Luc - Université catholique de Louvain). All participants signed an informed consent form and received financial compensation for their participation.

### Experimental design

**Figure 1:**
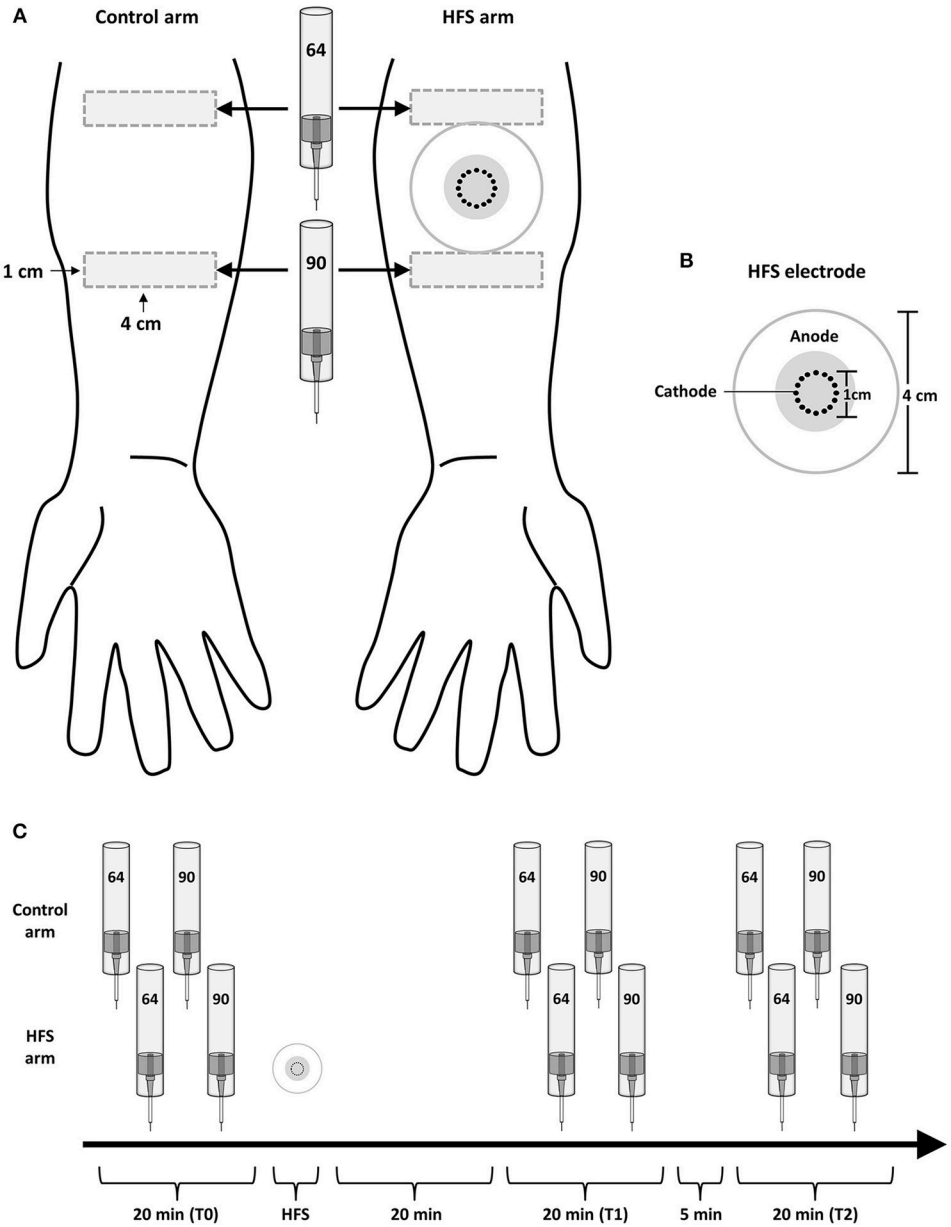
**Experimental setup**. **(A)** High frequency electrical stimulation of the skin (HFS) was applied to the left or right volar forearm. Two different intensities of pinprick stimulation (64 and 90 mN) were applied to the skin surrounding the area onto which HFS was applied as well as to the same skin area on the contralateral arm, which served as control. **(B)** The electrode used to deliver HFS consisted in 16 blunt stainless steel pins placed in a 10-mm diameter circle (cathode), surrounded by a concentrically-located stainless steel anode. **(C)** The effect of HFS on the responses elicited by the pinprick stimuli was assessed at three different time points: before HFS (T0), 20 min after HFS (T1) and 45 min after HFS (T2).

The design of the experiment is summarized in Figure 1. During the experiment, participants were comfortably seated in a chair with their arms resting on a table in front of them. Measurements were performed before (T0), 20 min after (T1) and 45 min after (T2) applying HFS to the left or right volar forearm. To avoid any confounding effect of handedness, the arm onto which HFS was applied (dominant vs. non-dominant) was counterbalanced across participants. Handedness was assessed using the Flinders Handedness Survey (Nicholls et al., 2013). For each measurement (T0, T1, T2), mechanical pinprick stimuli were applied to the skin adjacent to the area onto which HFS was delivered, and to the corresponding skin area of the contralateral arm which served as control. Two intensities of pinprick stimulation were used (64 and 90 mN). Each intensity was applied to a different skin site, distal or proximal from the site onto which HFS was delivered. This was balanced across participants, as well as the order of presentation of the two pinprick intensities, and the arm onto which stimuli were first applied (HFS vs. control arm).

### Induction of increased pinprick sensitivity

Transcutaneous high frequency electrical stimulation (HFS) was used to induce increased pinprick sensitivity in the surrounding unconditioned skin. The stimulation was applied to the volar forearm, 10 cm distal to the cubital fossa and consisted of five trains of 100 Hz (pulse width: 2 ms) lasting 1 s each. The time interval between each train was 10 s. The intensity of stimulation was individually adjusted to 20 × the detection threshold to a single pulse (0.28 ± 0.09 mA; mean ± sd). The electrical pulses were triggered by a programmable pulse generator (Master-8; AMPI Israel), produced by a constant current electrical stimulator (Digitimer DS7A, Digitimer UK), and delivered to the skin using a specifically designed electrode designed and built at the Centre for Sensory-Motor Interaction (Aalborg University, Denmark). The cathode consists of 16 blunt stainless steel pins with a diameter of 0.2 mm protruding 1 mm from the base. The 16 pins are placed in a circle with a diameter of 10 mm. The anode consists of a surrounding stainless steel ring having an inner diameter of 22 mm and an outer diameter of 40 mm.

### Mechanical pinprick stimulation

**Figure 2:**
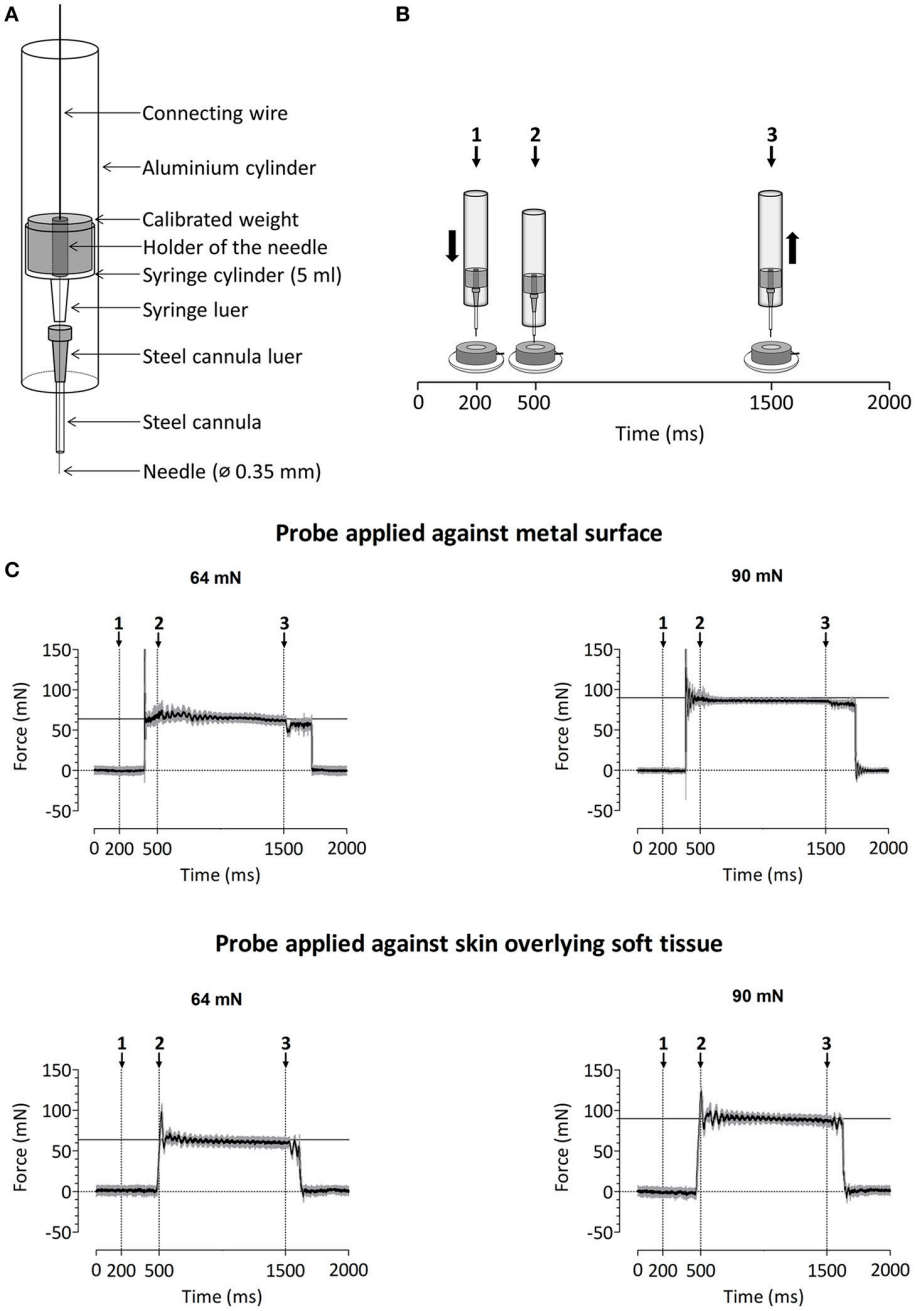
**(A)** Design of the custom-build mechanical pinprick stimulator used to record pinprick-evoked potentials. **(B)** Calibration protocol for the mechanical pinprick stimulus. Each trial started with a 200 ms baseline period. Subsequently, the probe, moved during 300 ms downwards to the force transducer. Then, the probe was maintained at this position for 1000 ms and withdrawn back to its initial position during 300 ms. A total of 30 trials were recorded. **(C)** Time course of the normal force generated against the force transducer with and without overlying soft tissue using the two different pinprick intensities (64 and 90 mN). The black line shows the average force across the thirty trials. The gray area shows the standard deviation.

A custom-built device was used to deliver calibrated mechanical pinprick stimuli (Figure 2A). The stimulator consists of a cylindrical stainless steel flat tip probe (diameter: 0.35 mm, uniform geometry) on top of which rests a calibrated cylindrical weight. The probe and weight are mounted inside an aluminum tube. When applied perpendicular to the skin, the probe and weight slide freely inside the tube, thereby maintaining a constant normal force entirely determined by the total mass of the probe and weight. To calibrate the pinprick stimulator, the device was attached to a Scara LS3 4-axis robot (DENSO Products and Services Americas, Inc., CA, USA), and a 6-axis strain-gauge force-torque transducer (Nano 43, ATI Industrial Automation, Inc., Apex, NC, USA) was used to measure the applied normal force with a precision of 1/256 N (Figure 2B). The time course of the normal force generated by applying and maintaining the needle against the force-torque transducer is shown in Figure 2C. Because the elastic properties of the skin and underlying soft tissues may be expected to significantly affect this time course (especially the initial increase in normal force), we also measured the normal force generated by applying the needle against a preparation of skin and muscle tissues taken from the thigh of a chicken (Figure 2C).

In a previous study conducted using a range of pinprick intensities (16–512 mN) and a constant 0.25 mm tip diameter, we found that the brain responses elicited at intermediate pinprick intensities (64 mN) showed the strongest enhancement after the induction of secondary hyperalgesia (van den Broeke et al., 2015). It has been suggested that the strength of activation of mechanical nociceptors is not related to the total applied normal force, but to the applied normal force relative to the circumference of the stimulation probe (Garell et al., 1996). In the present study, the tip diameter was 0.35 mm. Therefore, we used two different weights, one matching the total normal force (64 mN), and the other matching the ratio of normal force per circumference (90 mN, corresponding to 89 mN/mm). Each pinprick stimulus was delivered by applying the probe slowly onto the skin and moving the tube downwards and upwards with a total duration of approximately three seconds (see Supplementary Video 1). The reason for doing so is that the time course of the force applied onto the skin at contact onset is not only dependent on the weight of the probe, but also on the speed of the probe and hence its deceleration when contacting the skin. In order to minimize the peak of force at contact onset, the pinprick was applied onto the skin in a slowly fashion. For each intensity of pinprick stimulation (64 and 90 mN), each arm (HFS and control arm) and each time point (T0, T1, and T2), a total of 20 stimuli were administered. The stimuli were delivered using a random inter-stimulus interval ranging from 3 to 5 s (self-paced). To avoid sensitization of the stimulated skin, the target of the pinprick stimulus was displaced by the experimenter after each stimulus.

### EEG recording

The electroencephalogram (EEG) was recorded using 64 actively shielded Ag-AgCl electrodes mounted in an elastic electrode-cap and arranged according to the international 10–20 system (Waveguard64 cap, Advanced Neuro Technologies, The Netherlands). Participants were instructed to sit as still as possible and keep their gaze fixed on a black cross displayed at a distance of approximately 2.0 m. The EEG signals were amplified and digitized using a sampling rate of 1000 Hz and an average reference (HS64; Advanced Neuro Technologies, The Netherlands). Eye movements were recorded using two surface electrodes placed at the upper-left and lower-right sides of the left eye. Electrode impedances were kept below 20 kΩ. To generate a trigger in the EEG that marked the actual time at which the needle touched the skin we used a high resistance switch triggered by the change in impedance occurring between the probe and a ground electrode placed against the skin at the wrist. A thin layer of conductive gel was used to lower the impedance between the probe and the skin. The delay between the first contact of the skin and trigger generation was almost zero; on average (±SD) 0.046 ± 0.015 ms as tested in 40 trials using a shortcut circuitry with digitization of the trigger responses.

### Intensity of perception

The effect of HFS on the intensity of the percept elicited by mechanical pinprick stimulation was assessed by asking participants to rate the intensity of the stimuli on a numerical rating scale (NRS) ranging from 0 (no perception) to 100 (maximal pain), with 50 representing the transition from non-painful to painful domains of sensation. Within each block of 20 stimuli, the participants were asked to rate pseudo-randomly 10 out of the 20 stimuli directly after receiving the stimulus.

### Data analysis

#### Intensity of perception

To confirm the successful induction of increased pinprick sensitivity after HFS, we performed a General Linear Model (GLM) repeated measures ANOVA analysis on the intensity of perception ratings using two within-subject factors: *time* (T0, T1 and T2) and *treatment* (control vs. HFS arm) for both stimulation intensities (64 and 90 mN). The assumption of sphericity was tested using Mauchly's test of sphericity. In those cases where the data violated the assumption of sphericity, *F*-values were corrected using the Greenhouse-Geisser procedure. The level of significance was set at *p* < 0.05. For *post-hoc* tests, *p*-values were Bonferroni corrected for the number of tests. The statistical analyses were conducted using SPSS 18 (SPSS Inc., Chicago, IL, USA). The effect of HFS was assessed using the interaction between the factors *time* and *treatment*.

#### EEG preprocessing

The EEG signals were analyzed offline using Letswave 6.0 (www.nocions.org/letswave) and MATLAB 2014b (The MathWorks Inc., Natick, MA, USA). After applying a DC correction and a 0.3–30 Hz band pass zero-phase Butterworth filter to the continuous EEG recordings, the signals were segmented into epochs extending from −500 to +2000 ms relative to stimulus onset. Epochs contaminated by eye movements or eye blinks were corrected using an Independent Component Analysis (ICA; Jung et al., 2000). Denoised epochs were then re-referenced to linked mastoids (M1M2). After applying a baseline correction (reference interval: −500 to 0 ms), epochs with amplitude values exceeding ±100 μV were rejected as these were likely to be contaminated by artifacts. Finally, separate average waveforms were computed for each participant, time point (T0, T1, and T2), stimulation site (HFS and control) and stimulation intensity (64 and 90 mN). One subject was excluded because the EEG signals contained too many artifacts, which had as consequence that after artifact correction there were no trials left to compute an average waveform.

#### Pinprick evoked potentials (PEPs)

The effect of HFS on the EEG responses to pinprick stimulation was assessed using a spatio-temporal non-parametric cluster-based permutation approach (Maris and Oostenveld, 2007; Groppe et al., 2011). The advantage of this approach is that it is well suited to analyze long-lasting changes in EEG signals which cannot be summarized as a single peak having a given latency and amplitude, and it provides a simple way to solve the multiple comparison problem related to the point-by-point analysis of EEG signals (Maris and Oostenveld, 2007). First, we computed, for each subject and all electrodes, *difference* waveforms assessing the change in ERP waveform at T1 vs. T0 and T2 vs. T0 at the control arm (control arm_T1_ – control arm_T0_; control arm_T2_ – control arm_T0_) and at the HFS-treated arm (HFS arm_T1_ – HFS arm_T0_; HFS arm_T2_ – HFS arm_T0_). Then, we performed the cluster-based permutation test on the *difference* waveforms of both arms, thereby testing the *time x treatment* interaction. The test included the whole spatial (64 electrodes) and temporal (from 0 to 2000 ms after stimulus onset) dimensions of the dataset, and consisted of the following steps. First, the *difference* waveforms obtained at each electrode were compared by means of a point-by-point paired-sample *t*-test. Then, samples above the critical *t*-value for a parametric one-sided test that were adjacent in time and space were identified and clustered. Electrodes were considered as adjacent if their distance was < 0.42 cm, based on their standard Cartesian coordinates (Delorme and Makeig, 2004). Considering our 64 channel setup, most electrodes were considered to have four neighbors based on this criterion. An estimate of the magnitude of each cluster was then obtained by computing the sum of the absolute *t*-values constituting each cluster (cluster-level statistic). Random permutation testing (2000 times) of the subject-specific *difference* waveform of the two arms (performed independently for every subject) was then used to obtain a reference distribution of *maximum* cluster magnitude. Finally, the proportion of random partitions that resulted in a larger cluster-level statistic than the observed one (i.e., *p*-value) was calculated. Clusters in the observed data were regarded as significant if they had a magnitude exceeding the threshold of the 95th percentile of the permutation distribution (corresponding to a critical alpha-level of 0.05). This analysis was performed independently for both time points (T1 and T2) and stimulation intensities (64 and 90 mN).

## Results

### Intensity of perception

**Figure 3:**
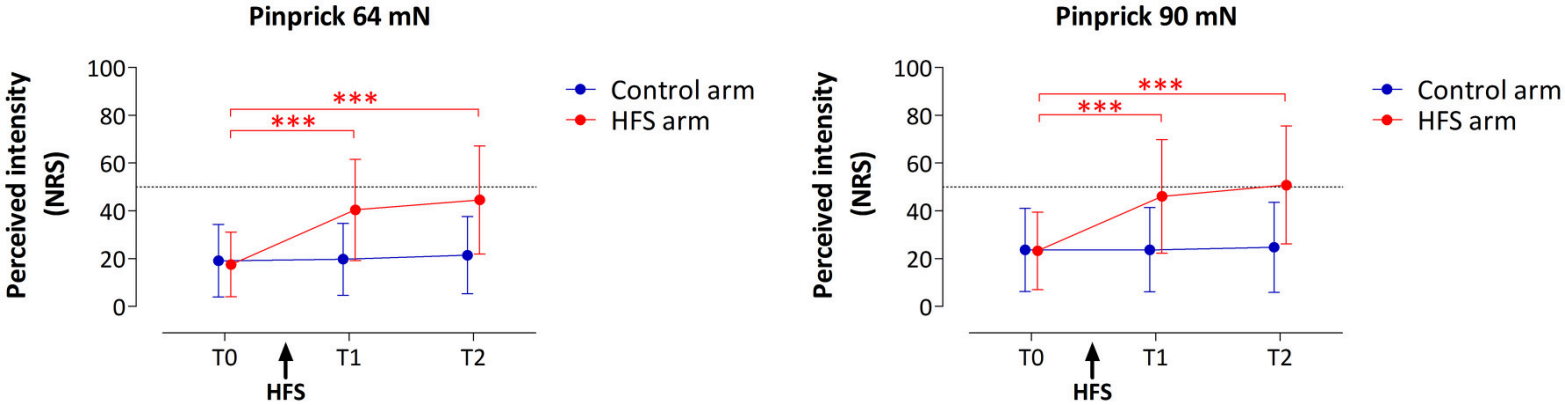
**Effect of HFS on intensity of perception elicited by the two pinprick intensities (64 and 90 mN)**. Shown are the group-level mean and standard deviation of the numeric rating scale (NRS) scores obtained at the three different time points: before HFS (T0), 20 min after HFS (T1) and 45 min after HFS (T2). Asterisks denote a statistically significant increase of the HFS treated arm compared to T0 (*p* < 0.001, *post-hoc* paired *t*-test).

HFS induced a clear increase in pinprick sensitivity on the treated arm, as shown by the changes in the intensity of the percept elicited by 64 and 90 mN pinprick stimulation (Figure 3). This was confirmed by repeated-measures ANOVA, which showed a significant *time* × *treatment* interaction for both the 64 mN stimulus [*F*_(2, 36)_ = 31.388, *p* < 0.001, η^2^ = 0.636] and the 90 mN stimulus [*F*_Greenhouse−Geisser (1.16, 20.87)_ = 24.494, *p* < 0.001, η^2^ = 0.576].

For both pinprick intensities the univariate within-subject contrasts showed that intensity of perception was significantly enhanced at the HFS-treated arm, both at T1 [*64 mN*: *F*_(1, 18)_ = 37.962, *p* < 0.001, η^2^ = 0.678; *90 mN*: *F*_(1, 18)_ = 25.421, *p* < 0.001, η^2^ = 0.585] and at T2 [*64 mN*: *F*_(1, 18)_ = 38.131, *p* < 0.001, η^2^ = 0.679; *90 mN*: *F*_(1, 18)_ = 26.012, *p* < 0.001, η^2^ = 0.591]. *Post-hoc* tests showed, for both intensities, a statistically significant increase of perceived intensity at the treated arm, both at T1 [*64 mN*: paired *t*-test; *t*_(18)_ = −6.436, *p* < 0.001; *90 mN*: *t*_(18)_ = −5.060, *p* < 0.001] and at T2 [*64 mN*: *t*_(18)_ = −6.402, *p* < 0.001; *90 mN*: *t*_(18)_ = −5.245, *p* < 0.001].

### Pinprick evoked brain potentials (PEPS)

#### PEPs elicited by 64 mN pinprick stimulation

**Figure 4:**
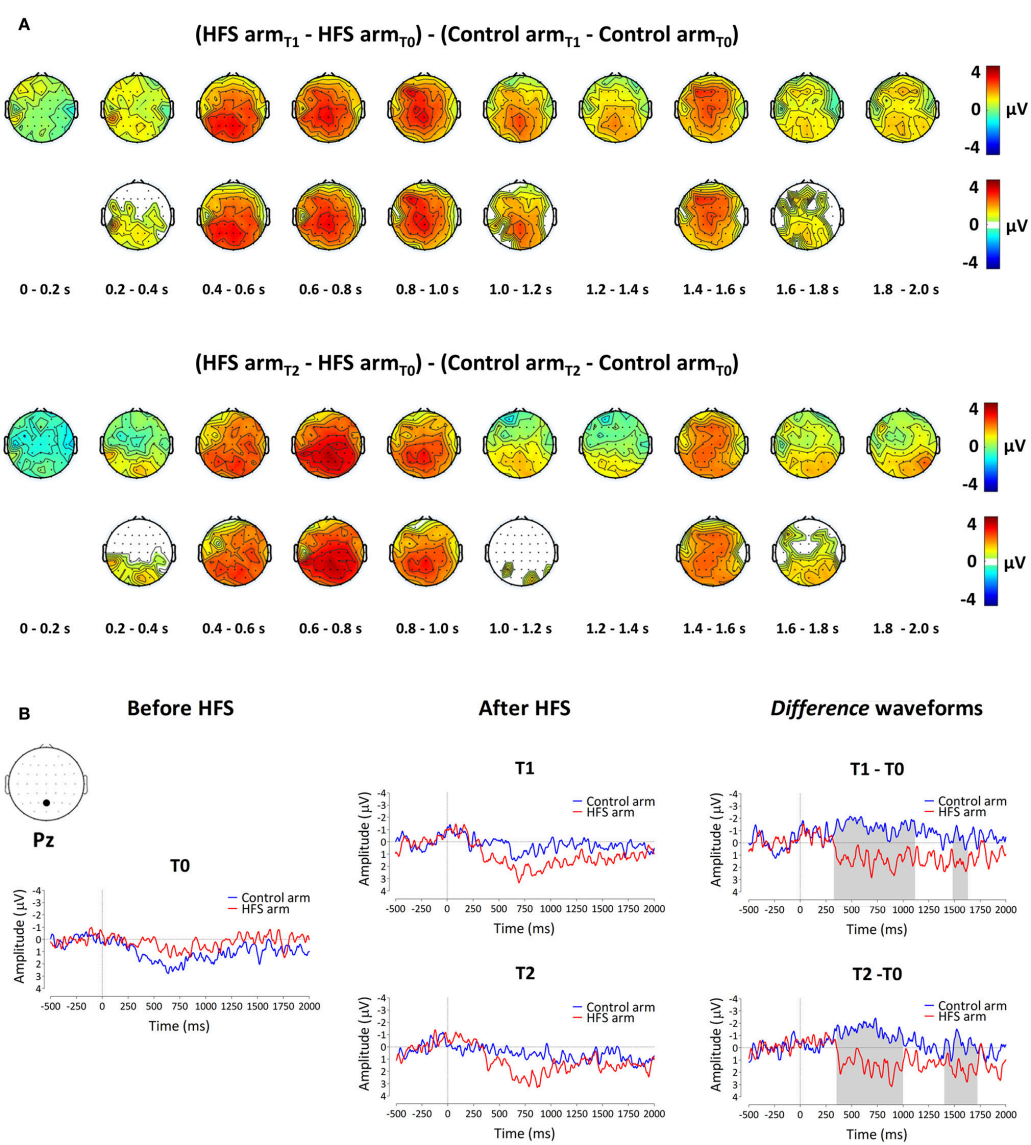
**The effect of HFS on PEPs elicited by 64 mN pinprick stimulation**. **(A)** The first row of scalp topographies shows the temporal evolution of the group-level average topography of the difference between the subtracted waves (T1 minus T0) of both arms for T1. Red denotes an increase of the ERP amplitude at the HFS arm compared to the control arm whereas blue indicates a decrease of ERP amplitude at the HFS arm compared to the control arm. Each topographic plot corresponds to the average amplitude within successive segments of 200 ms. The second row of scalp topographies shows the same topographies, masked by the spatiotemporal pattern of the significant clusters identified using the spatiotemporal cluster-based permutation testing. Masked means that only the time-electrode samples included in the significant clusters are displayed. The third row of scalp topographies shows the temporal evolution of the group- level average topography of the difference between the subtracted waves (T2 minus T0) of both arms for T2. The fourth row of scalp topographies shows those topographies masked using the spatiotemporal pattern of the significant clusters. Scalp topographies were corrected (flipped) according to the side of HFS stimulation. **(B)** Group-level average ERP waveforms of the signals measured from Pz vs. M1M2, before HFS (T0), 20 min after HFS (T1) and 45 min after HFS treatment (T2) and group-level average difference waveforms (T1–T0 and T2–T0) for the control arm (blue) and the HFS-treated arm (red). Gray shadings indicate the time intervals of the significant clusters shown in **(A)**.

The group-level average waveforms of the PEPs elicited by the 64 mN stimulation are shown in Figure 4. The elicited response consisted mainly of a long-lasting positive wave, maximal at midline central-posterior electrodes. The results of the cluster-based permutation test performed on the ERP *difference* waveforms (after vs. before HFS) of both arms (control vs. HFS) for both time points (T1 and T2) revealed two positive clusters having a *p*-value smaller than the critical alpha level of 0.05, both at T1 and at T2 (Figure 4). At T1, the first cluster extended between 326 and 1122 ms after stimulus onset (*p* < 0.001), and the second cluster extended between 1481 and 1638 ms (*p* < 0.05). At T2, the first cluster extended between 350 and 1005 ms (*p* < 0.001), and the second cluster extended between 1398 and 1728 ms (*p* < 0.05). Both at T1 and at T2, the first cluster was widely distributed but maximal at central-posterior scalp electrodes, whereas the second cluster displayed a more central-frontal distribution (Figure 4A). These two clusters demonstrate a statistically significant difference in PEP amplitude between the two arms, which was present both 20 min (T1) and 45 min (T2) after applying HFS.

**Figure 5:**
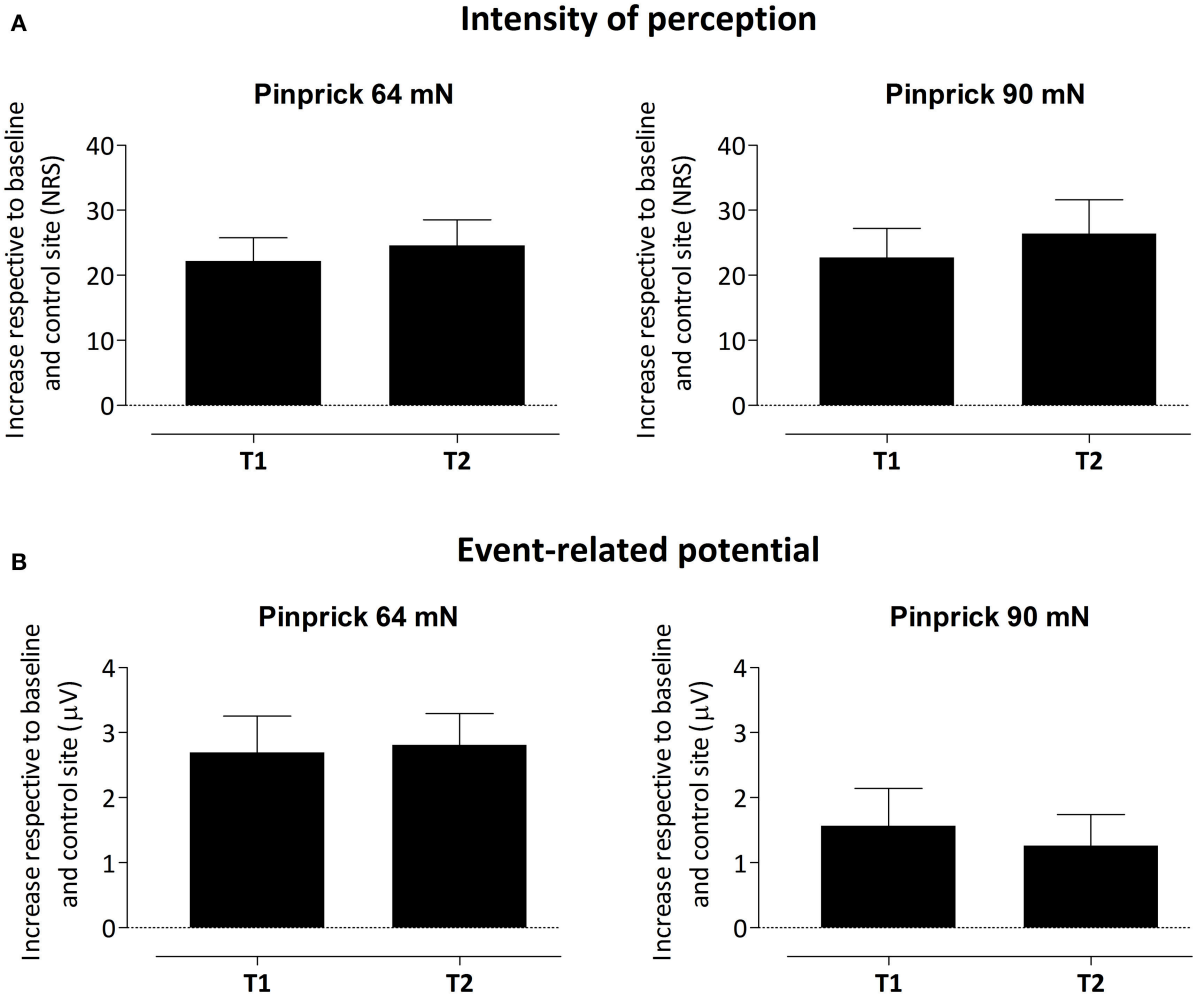
**Average (and SEM) group-level increase of the intensity of perception (A) and event-related potential amplitude, i.e., mean value of cluster 1 (B) respective to baseline and control site for both T1 and T2**.

To assess whether the difference in PEP amplitude between the two arms after HFS was due to an *increase* of PEP amplitude at the HFS-treated arm, a *decrease* of PEP amplitude at the control arm, or *both*, we performed *post-hoc* tests (paired *t*-tests, two-sided, Bonferroni corrected) on the individual mean amplitude values calculated within cluster 1 at electrode Pz. The paired *t*-tests revealed both (1) at the HFS-treated arm, a significant *increase* in mean PEP amplitude at T1 compared to T0 [*t*_(18)_ = −3.088, *p* = 0.006] and T2 compared to T0 [*t*_(18)_ = −3.024, *p* = 0.007] and (2) at the control arm, a significant *decrease* in mean PEP amplitude at T1 compared to T0 [*t*_(18)_ = 3.432, *p* = 0.003] and at T2 compared to T0 [*t*_(18)_ = 3.133, *p* = 0.006]. Figure 5 shows the average (and SEM) group-level increase in perceived intensity and ERP amplitude (mean value cluster 1) respective to baseline and control site for T1 and T2.

#### PEPs elicited by 90 mN pinprick stimulation

**Figure 6:**
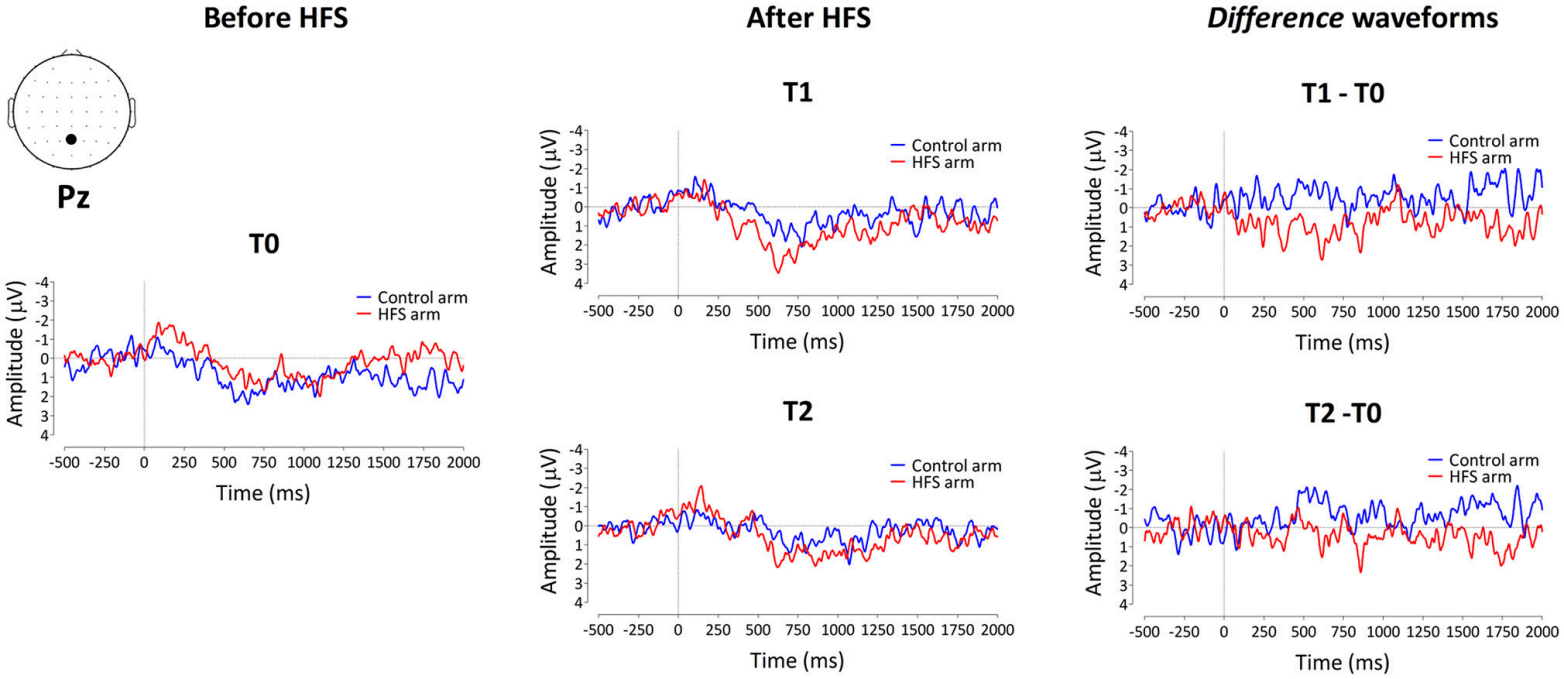
**Group-level average ERP waveforms measured from Pz vs. M1M2, before HFS (T0), 20 min after HFS (T1) and 45 min after HFS treatment (T2), as well as the difference waveforms, for both the control arm (blue) and HFS-treated arm (red) for the 90 mN pinprick intensity**.

The group-level average waveforms of the PEPs elicited by 90 mN stimulation are shown in Figure 6. Such as for the 64 mN stimulus, the elicited response consisted mainly of a long-lasting positive wave, maximal at central-posterior electrodes. The permutation testing did not identify any cluster having a *p*-value smaller than the critical alpha level of 0.05.

## Discussion

The present study shows, for the first time, that the increase in brain response elicited by the 64 mN mechanical pinprick stimulation in the area of secondary hyperalgesia follows the same time course as the increase in pinprick sensitivity, both being present 20 and 45 min after applying HFS.

### T2: a crucial time point

Performing a second post measurement 45 min after applying HFS is crucial as it allows distinguishing *unspecific short-lasting* from *specific long-lasting* effects of HFS. The increase in PEP positivity followed the long-lasting time course of the increase in pinprick sensitivity. Indeed, both were present 20 and 45 min after HFS. Importantly, this long-lasting increase in PEP positivity is in striking contrast with the short-lasting enhancement of EEG responses to brief non-nociceptive vibrotactile and thermal stimulation that have been reported in previous studies (van den Broeke and Mouraux, 2014a,b). Indeed, in these studies, the EEG enhancements were observed 20 min after HFS, but disappeared completely 45 min after HFS. The fact that HFS appears to produce various effects with distinct time courses suggests the existence of multiple mechanisms. In addition to a long-lasting central sensitization of mechanical nociceptive pathways, leading to a selective increase in pinprick sensitivity, HFS appears to also produce short-lasting and modality unspecific changes, which could be the consequence of higher-order cognitive mechanisms. For example, delivering HFS on one arm could lead to a sustained orientation of spatial attention toward the treated arm.

### Increased pinprick sensitivity

Such as in our previous studies (van den Broeke and Mouraux, 2014a,b), the perception elicited by the pinprick stimuli at baseline were not qualified as painful. Therefore, the increased pinprick sensation observed after applying HFS does not satisfy the current definition of “secondary hyperalgesia” proposed by the International Association for the Study of Pain (IASP), which restricts the term hyperalgesia to “increased pain from a stimulus that normally provokes pain” (Loeser and Treede, 2008). However, studies using microneurography have clearly demonstrated that punctate probes like the ones used in the present study preferentially activate mechanosensitive nociceptors when applied against the skin (Garell et al., 1996; Slugg et al., 2000, 2004). Moreover, a study comparing the perceptual pain thresholds in human volunteers with the thresholds for nociceptors in animals using the same mechanical punctate probes, suggests that the non-painful sharp pricking sensation is mediated by mechanosensitive nociceptors (Greenspan and McGillis, 1991). Therefore, even though the mechanical stimuli elicited, at baseline, a pinprick percept that was not qualified as painful, the increased perception to these stimuli when delivered in the area of increased pinprick sensitivity is most probably related to a central sensitization of mechanical nociceptive pathways.

### PEPs elicited from the area of increased pinprick sensitivity

In our previous study, a range of pinprick intensities (16–512 mN) was used to characterize the effect of stimulation intensity on the PEPs elicited by stimulation before vs. after and within vs. outside the area of secondary hyperalgesia (van den Broeke et al., 2015). We observed a nonlinear (inverted U-shape) relationship between stimulation intensity and the increase in PEPs elicited from the area of secondary hyperalgesia. The strongest (and only significant) increase in PEP was elicited by the intermediate 64 mN stimulation intensity. The current results are in agreement with that study. Indeed, we observed a significant increase in PEP for the 64 mN stimulation, but not for the 90 mN stimulation. At present, we can only speculate about why there is no significant increase in PEP amplitude for the 90 mN and higher stimulation intensities. One possibility could be a ceiling effect: at 90 mN, the PEPs recorded in the absence of sensitization could reach an upper limit above which no further increase in PEP magnitude can be observed. Another possibility could be that the higher levels of arousal elicited by higher stimulation intensities could, in some way, inhibit the cortical responses underlying the increase in PEP magnitude. Supporting this hypothesis, a previous study observed a similar non-linear relationship between the enhancement of a positive wave elicited by known vs. novel images and different levels of arousal in an emotional recognition task. The largest difference in amplitude, peaking around 300 ms after stimulation onset, was observed for intermediate levels of arousal (Schaefer et al., 2009).

## Author contributions

EV, JL, and AM were involved in the conception and design of the study. EV, JL, and GH collected and analyzed the data. EV, JL, GH, and AM interpreted the data and drafted the manuscript. All authors have approved the final version of the manuscript.

## Funding

EV is supported by the Belgian National Foundation for Scientific Research (Mandat d'impulsion scientifique FNRS 14613969) and the ERC “Starting Grant” (PROBING PAIN 336130). AM is supported by the ERC “Starting Grant” (PROBING PAIN 336130). GH is supported by the Belgian National Foundation for Scientific Research (Mandat d'impulsion scientifique FNRS 14613969), and JL is supported by the ERC “Starting Grant” (PROBING PAIN 336130).

### Conflict of interest statement

The authors declare that the research was conducted in the absence of any commercial or financial relationships that could be construed as a potential conflict of interest.

## Supplementary material

Supplementary Video 1 **Demonstration pinprick stimulation**.
